# Biodegradation of Ochratoxin A for Food and Feed Decontamination

**DOI:** 10.3390/toxins2051078

**Published:** 2010-05-13

**Authors:** Luís Abrunhosa, Robert R. M. Paterson, Armando Venâncio

**Affiliations:** IBB, Institute for Biotechnology, Bioengineering, Centro de Engenharia Biológica, Universidade do Minho, Campus de Gualtar, 4710-057 Braga, Portugal; Email: russell.paterson@deb.uminho.pt (R.R.M.P.); avenan@deb.uminho.pt (A.V.)

**Keywords:** ochratoxin A, biodegradation, detoxification, decontamination

## Abstract

Ochratoxin A (OTA) is one of the most important mycotoxins that is found in food and feed products. It has proven toxic properties, being primarily known for its nephrotoxicity and carcinogenicity to certain animal species. OTA is produced by several species of *Aspergillus *and *Penicillium* that can be found in a wide variety of agricultural products, which makes the presence of OTA in these products common. Many countries have statutory limits for OTA, and concentrations need to be reduced to as low as technologically possible in food and feed. The most important measures to be taken to control OTA are preventive in order to avoid fungal growth and OTA production. However, these measures are difficult to implement in all cases with the consequence of OTA remaining in agricultural commodities. Remediation processes are often used to eliminate, reduce or avoid the toxic effects of OTA. Biological methods have been considered increasingly as an alternative to physical and chemical treatments. However, examples of practical applications are infrequent. This review will focus on the (i) known microorganisms and enzymes that are able to biodegrade OTA; (ii) mode of action of biodegradation and (iii) current applications. A critical discussion about the technical applicability of these strategies is presented.

## 1. Introduction

### 1.1. Overview

The discovery of aflatoxins in the 1960s [1], with approximately 100,000 turkey poult deaths in England, was the seminal event that made the scientific community realize that mold secondary metabolites could be responsible for food and feed safety problems. Several other mycotoxins were identified when fungi in food were more fully investigated. Ochratoxin A (OTA) was purified and characterized from *Aspergillus ochraceus* Wilh. strain K‑804 [2,3] isolated from sorghum grain, and proved to be acutely toxic to Pekin ducklings, mice and rats [4]. Nowadays, OTA is one of the most relevant mycotoxins, with its presence in food and feed products being regulated in many countries.

**Figure 1:**
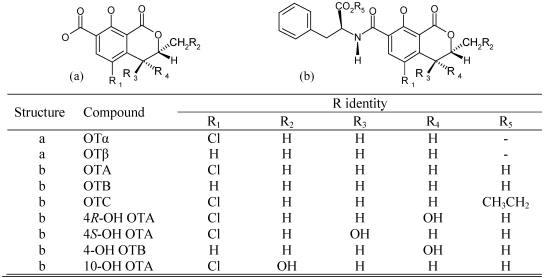
Molecular structure of ochratoxins naturally produced by filamentous fungi [2,5,6].

OTA is composed of a 7-carboxy-5-chloro-8-hydroxy-3,4-dihydro-3-*R*-methylisocoumarin (ochratoxin α) moiety and a L-*β*-phenylalanine molecule, which are linked through the 7‑carboxy group by an amide bond. The compound belongs to a group of fungal secondary metabolites, commonly known as ochratoxins (Figure 1), which share a similar chemical structure. Ochratoxin B (OTB), ochratoxin C (OTC), ochratoxin α (OTα), ochratoxin β (OTβ), (4‑*R*)- and (4‑*S*)-hydroxy-ochratoxin A (4*R*‑OH OTA and 4*S*‑OH OTA), 4-hydroxy-ochratoxin B (4‑OH OTB) and 10-hydroxy-ochratoxin A (10‑OH OTA) are naturally produced by certain fungi.

Of these, OTA is the predominant compound detected in agriculture commodities and the most relevant to food and feed safety. The IUPAC formula is L‑phenylalanine-*N*-[(5-chloro-3,4-dihydro-8-hydroxy-3-methyl-1-oxo-1*H*-2-benzopyran-7-yl)carbonyl]-(*R*)-isocoumarin and its chemical abstract specification (CAS) is 303-47-9 [7]. OTA is a colorless crystal of empirical formula C20H18O6NCl, which has a molecular weight of 403.822 Da [8]. The melting points are 94-96 °C and 169 °C when crystallized from benzene or xylene, respectively. Maximum absorbances in (a) 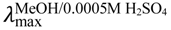 are 216, and 330 nm (ε 37,060 and 6050, respectively); and (b) 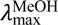 are 214 and 332 nm (ε 37,200 and 6330, respectively) [9]. The infrared spectrum in chloroform presents major peaks at 3380, 1723, 1678 and 1655 cm^−1^. OTA has weak acid properties with pKa_1_ 4.2-4.4 and pKa_2_ 7.0-7.3, from the carboxyl group of phenylalanine and from the phenolic hydroxyl group of the isocoumarin, respectively [7]. OTA produces a green-bluish fluorescence when excited by UV light (366 nm) using TLC, which changes to a dark blue fluorescence when exposed to ammonia vapors, aqueous NaHCO_3_ or NaOH [10]. The fluorescence properties are commonly used for detection and identification purposes in TLC and HPLC. LC-MS can be employed to detect OTA. The identity of OTA can be confirmed by converting it to the methyl ester [11], and/or to OTα using carboxypeptidase A. OTA is highly soluble in polar organic solvents, soluble in aqueous sodium hydrogen carbonate and slightly soluble in water.

### 1.2. Fungi

OTA is produced by several *Aspergillus *species and *Penicillium verrucosum* and *P. nordicum*. These penicillia are the species most authoritatively listed as OTA-producing. The *P. viridicatum* strains that were reported as OTA producers were reclassified as *P. verrucosum* by Pitt [12]. Other species such as *P. chrysogenum*, *P. brevicompactum*, *P. crustosum*, *P. olsonii* and *P. oxalicum* have been claimed as OTA producers [13,14]. Nevertheless, a careful confirmation of these findings is required since no other authors report the capacity of these species to produce OTA. Strains of a larger number of species are known to produce OTA in *Aspergillus*. Species accepted by Frisvad and co-authors [15] are listed in Table 1.

**Table 1:** OTA producing fungi.

Fungi species	References
***Aspergillus *section *Circumdati***	
*A. cretensis*	[16]
*A. flocculosus*	[16]
*A. melleus*	[17]
*A. ochraceus*	[3,16]
*A. ostianus*	[17]
*A. persii*	[18]
*A. petrakii*	[17]
*A. pseudoelegans*	[16]
*A. roseoglobulosus*	[16]
*A. sclerotiorum*	[16,17,19]
*A. steynii*	[16]
*A. sulphureus*	[16,17,19]
*A. westerdijkiae*	[16]
***Aspergillus *section *Flavi***	
*A. alliaceus* (*Petromycesalliaceus*)	[19,20]
*Petromyces albertensis*	[15]
***Aspergillus *section *Nigri***	
*A. carbonarius*	[21,22,23]
*A. lacticoffeatus*	[23]
*A. niger*	[23,24]
*A. sclerotioniger*	[23]
***Penicillium***	
*P. nordicum*	[25]
*P. verrucosum*	[12,26,27]

Some other species were reported incorrectly as OTA producers, due to either misidentification of the isolates in question or to the use of contaminated cultures. Others still were reclassified, having now a different scientific name. For example, the original *A. ochraceus* strain from which OTA was first isolated, was reclassified as *A. westerdijkiae*, and many other *A.ochraceus* strains as *A. steynii* [16]. 

The major OTA producers in food and feed products are considered to be *A. alliaceus*, *A. carbonarius*, *A. ochraceus*, *A. steynii*, *A. westerdijkiae*, *P. nordicum* and *P. verrucosum* [15]. These are mainly associated with agricultural crops pre-harvest, or in post harvest storage situations. *P. nordicum* is a high OTA producer and is isolated predominantly from certain cheeses and fermented meats. *Aspergillus niger*, which is a very common species, is less relevant since most of the isolates are non-ochratoxigenic [28,29] and the others usually produce small amounts of OTA [30]. However, since it is a widely used species in several biotechnological processes it can pose some safety issues in the manufacture of food grade organic acids and enzymes. The other OTA producers are rare and do not pose a great concern for food safety.

### 1.3. Biosynthetic pathway

The OTA biosynthesis pathway is not completely established. However, it is known that the isocoumarin is a pentaketide which derives from the polyketide pathway and that L-*β*-phenylalanine derives from the shikimic acid pathway. More precisely, experimental studies with radioactive labeled precursors demonstrated that the isocoumarin is synthesized by head-to-tail condensation of five acetate units with the subsequent addition in C7 of a unit of methionine, which is subsequently oxidized to carboxyl [31,32,33]. Apparently, L-*β*-phenylalanine is coupled unaltered to the isocoumarin moiety since an isolated *A. ochraceus* enzyme fraction was able to link the two portions of the molecule [31]. Finally, the chlorine is incorporated in C5 from NaCl (although it is not known at what exact point of the biosynthesis) and probably through the action of chloroperoxidases [34]. There is little information about the genes responsible for these biosynthetic steps. Only DNA fragments that encode for some OTA polyketide synthases in *P. nordicum* [35], *A. ochraceus* [36] and *A. carbonarius* [37] have been identified.

### 1.4. Physiology of OTA production

OTA production depends on factors such as temperature, substrate water activity (aw), and micronutrients [9]. The optimal conditions for OTA production by *A. carbonarius*, *A. ochraceus* and *P. verrucosum *are 15-20 °C and 0.95-0.98 aw [38]; 25-30 °C and 0.98 aw [39]; and 24 °C and 0.95-0.99 aw [40], respectively. The culture media most used for its biosynthesis are Yeast Extract Sucrose (YES) and Czapek Yeast Autolysate (CYA) [41].

### 1.5. Presence in commodities

OTA has been detected in a wide variety of agricultural commodities, livestock products and processed food (Table 2). Concentrations found in the final food products are lower than those found in raw materials since some processing steps can contribute actively to its elimination. For example, (i) malting; (ii) malt fermentation; (iii) white bread and (iv) whole-bread production can contribute to reduce OTA by 56% [42]; 21% [43]; 80% [44] and 40% [44], respectively. Additionally, reductions of 35%, 71% and 83% for mild, medium and strong coffee roasting, respectively, were reported [45]. Finally, the wine-making process contributes to almost 90% reduction of OTA [46].

**Table 2:** OTA levels found in some agricultural commodities, livestock products, processed food products.

Food products	Contamination levels	References
Beans	0.25-0.92 µg/Kg	[47]
Cocoa beans	0.35-14.8 µg/Kg	[48]
Corn	0.11-0.15 µg/Kg	[49]
Dried figs	<0.1-35.1 µg/Kg	[50]
Dried fruits	0.1-30 µg/Kg	[51]
Grapes	0.008-1.6 µg/Kg	[52]
Green coffee beans	0-48 µg/Kg	[53]
Milk	0.011-0.058 µg/L	[54]
Pork kidneys	0-15 µg/Kg	[55]
Pork meat	0-2.9 µg/Kg	[55]
Raisins	0.2-53.6 μg/Kg	[56]
Rice	1.0-27.3 µg/Kg	[57]
Spices	4.2-103.2 µg/Kg	[58]
Wheat, Barley, oats	0.1-17.8 µg/Kg	[59]
Wheat, oats and rye	0.03-27 µg/Kg	[60]
Baby food	0.06-2.4 µg/Kg	[61]
Beer	<0.01-0.135 µg/L	[62]
Breakfast cereals	0.4-8.8 µg/Kg	[63]
Cocoa products	0.22-0.77 µg/Kg	[64]
Grape juice	<0.003-0.311 µg/L	[65]
Pork products	<0.03-10.0 µg/Kg	[66]
Roasted coffee	3.2-17.0 µg/Kg	[67]
Salami	<0.006-0.40 µg/Kg	[68]
Wine	<0.003-0.388 µg/L	[65]

OTA is also detected in feed (Table 3) with the concentrations usually being higher than those in food. Processing steps such as extrusion may contribute to reduce concentrations [69]. Nevertheless, other practices can increase OTA concentrations. For example, some by-products derived from cereal processing, such as cracked grains, cereal cleanings, wheat and corn bran, are often the fractions most contaminated with OTA, and are usually directed for feed proposes [70]. Moreover, it is common practice to direct the lower contaminated commodities for human consumption while the most contaminated are used for feed.

**Table 3:** OTA levels found in some feed products and raw materials.

**Feed products**	**OTA levels average (µg/Kg)**	**References**
Poultry feed	0.5	[71]
Bovine feed	0.55	
Corn grains	3.95	
Corn gluten	1.95	
Cotton seed	6.19	
Palm kernel	3.19	
Poultry feed	27	[72]
Pig feed	34	
Rabbit feed	21.8	
Groundnut cake	50-100	[73]
Millets	>100	
Rice bran	10-100	
Sorghum	10->100	
Sunflower	30-49	

### 1.6. Toxicity

OTA is the most toxic and relevant of the known ochratoxins. However, some consider that OTC is equally toxic to OTA [74,75] since, when ingested, it is converted rapidly into OTA, which becomes available in the bloodstream [76,77]. OTA is known primarily for its nephrotoxicity. It was nephrotoxic for all tested animals with the exception of adult ruminants [78] and it appears to be the cause of porcine nephropathy, human Balkan endemic nephropathy (BEN) and chronic interstitial nephropathy (CIN) in North Africa [79,80,81]. OTA is also classified as possibly carcinogenic to humans (group 2B) since there is evidence for experimental animals but not for humans [82]. In addition, OTA has mutagenic, teratogenic, neurotoxic, hepatotoxic and immunotoxic properties [80]. Oral LD_50_ values are 1.0-6.0 mg/kg for pigs, 20-30 mg/kg for rats and 48-58 mg/kg for mouse [83]. In these studies, OTA also caused haemorrhages in almost all vital organs, nephrosis, and necrosis in the liver and lymphoid tissues. OTA is considered to be a cumulative toxic compound since it is easily absorb through the stomach and the small intestine but hardly eliminated through the biliary and urinary routes. Oral OTA half-lives are 35.5 days for humans, 21 days for monkeys, 72-120 hours for pigs, 55-120 hours for rats and 40 hours for mice [83]. The high elimination half-lives observed in some species are due to the strong OTA affinity to serum proteins, which limit its transfer from the blood to the hepatic and renal cells. OTA affinity to bovine serum albumin [84] and to human serum albumin [85] was observed *in vitro*, and its relationship with OTA excretion confirmed by Kumagai [86] who verified that albumin-deficient rats were able to eliminate this mycotoxin more quickly through urine.

OTα toxicity has not been so extensively studied. Nevertheless, some studies indicated it is essentially non-toxic. For example, OTα was ineffective as an immunosuppressor when tested in mice [87] and was considered 1,000-times less toxic than OTA in brain cells cultures [88]. Furthermore, OTα has an elimination half-live of 9.6 h in rats, which is well below that of OTA (103 h) [77]. Therefore, processes that lead to the conversion of OTA into OTα contributes substantially to reduce OTA toxic effects and, hence, are considered to be routes for OTA detoxification. 

A tolerable daily intake (TDI value) of 5 ng OTA/kg bw/day is recommended by the World Health Organization since it has toxic effects and is found in human blood [80,89,90,91] and in breast milk [80,92,93,94], thus proving human exposure. Furthermore, it is recommended that OTA levels in food and feed should be reduced as much as technologically possible. 

### 1.7. Elimination strategies

Several strategies can be employed to reduce OTA levels. The most important are preventive since they avoid the contamination of commodities in the first place. The use of (i) good agricultural practices, (ii) fungal resistant crop varieties, (iii) the correct application of fungicides and (iv) the proper storage of commodities are common measures that could, ideally, be part of a Hazard Analysis Critical Control Point (HACCP) scheme to minimize OTA at critical control points of the food supply chain.

However, fully implemented HACCP schemes are rare, and when the individual measures fail or are not in place, OTA remains in food and feed products. Decontamination or detoxification procedures can be used to remove or to reduce OTA levels. These measures, which are technologically diverse, are usually classified into physical, chemical or biological [95]. Physical methods consist of segregation, sorting, cleaning, peeling and shelling processes that aim to remove the most contaminated fractions of the commodities. They also may involve the utilization of sorbents as nutritional additives that absorb OTA hence reducing bioavailability. Chemical methods consist of the utilization of compounds to destroy OTA: some processes use ammonium (ammoniation), alkaline hydrolysis (nixtamalization), bisulphites and ozone (ozonation). These are reported generally as effective in the elimination of OTA and other mycotoxins [96]. However, the toxicological safety of the final product is not always guaranteed since some chemical residues may remain in products and the toxicity of the reaction products formed is not usually studied. Furthermore, there is a significant reduction in palatability and nutritive quality of treated products. 

Biological methods use microorganisms, which can decompose, transform or adsorb OTA to detoxify contaminated products or to avoid the toxic effects when mycotoxins are ingested. These are the technologies of choice for decontamination proposes because they present several advantages from being mediated by enzymatic reactions. For example, they are very specific, efficient, environmentally friendly, and they preserve nutritive quality. However, the non-pathogenicity of the microorganism and the non-toxicity of the reaction products formed are essential [97]. More research is needed to render these methods practical, effective and economically feasible.

## 2. Biodegradation of Ochratoxin A

**Figure 2:**
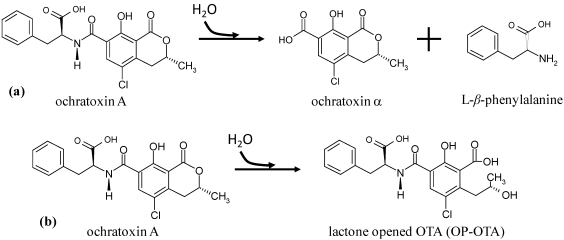
Ochratoxin A biodegradation pathways. (**a**) Amide bond hydrolysis of OTA; (**b**) Lactone ring hydrolysis of OTA.

Numerous microorganisms capable of degrading, adsorbing and detoxifying OTA are reported in the literature and some practical processes have been developed. Reports about the capacity of proteolytic enzymes to hydrolyze OTA can also be found. Two pathways may be involved in OTA microbiological degradation. First, OTA can be biodegraded through the hydrolysis of the amide bond that links the L-*β*-phenylalanine molecule to the OTα moiety (Figure 2a). Since OTα and L-*β*-phenylalanine are virtually non-toxic, this mechanism can be considered to be a detoxification pathway [97]. Second, a more hypothetical process involves OTA being degraded via the hydrolysis of the lactone ring [97]. In this case, the final degradation product is an opened lactone form of OTA (Figure 2b), which is of similar toxicity to OTA when administered to rats [74,77]. However, it is less toxic to mice and *Bacillus brevis* [74]. Although this is hypothetical, it is likely to occur since microbiological lactonohydrolases, which undertake a similar transformation, are common [98].

### 2.1. Microorganisms which degrade ochratoxin A

Several protozoal, bacterial, yeast and filamentous fungal species are able to biodegrade OTA. 

#### 2.1.1. Protozoa

Ruminants are able to biodegrade OTA following the pathway that yields phenylalanine and OTα [99] with rumen protozoa being primarily responsible [100]. Indeed, ruminants are resistant to OTA toxic effects because of this detoxification capability [101]. More evidence is provided when high concentrate-rich diets are fed to animals, which reduce the natural protozoan population of the rumen, leading to increased OTA absorption and accumulation in the blood of animals and excretion into the milk of cows, with resulting carryover to humans [102].

#### 2.1.2. Bacteria

Several bacteria were reported to degrade OTA. *Phenylobacterium immobile* degraded OTA present in a culture medium containing 0.1 mg OTA/L after three to five hours of incubation at 25 °C [103]. Four degradation products were identified, including OTα, which was not further metabolized. The proposed degradation pathway involved (i) a dioxygenase attack on the phenylalanine moiety; (ii) subsequent dehydrogenation to a catechol derivative that undergoes a meta-ring cleavage and (iii) a hydroxylation step that releases OTα. The authors verified that there was no growth with 200 mg OTA/L as the sole carbon and energy source. 

*Acinetobacter calcoaceticus* removed 0.1005 and 0.0636 mg OTA/L/h when grown at 25 °C and 30 °C, respectively, with 10 mg OTA/L [104]. OTα was detected, and it was assumed that the biodegradation pathway involved the hydrolysis of the peptide bond. Total elimination of OTA was achieved after five days at 30 °C. *Bacillus licheniformis* degraded 92.5% of 5 mg OTA/L after 48 h at 37 °C and OTα was detected [105]. Also, *Streptococcus salivarius* subsp. *thermophilus*, *Lactobacillus delbrueckii* subsp. *bulgaricus* and *Bifidobacterium bifidum* eliminated OTA present in milk samples with 0.05 and 0.1 mg OTA/L [106]. However, the detection of OTα was not reported in this study, which implies that adsorption rather than biodegradation may have occurred. A similar situation was reported in other studies: some *Bacilli* and *Lactobacillus* strains were demonstrated to eliminate 0.05 mg OTA/L added to culture medium-in particular, *L. bulgaricus*, *L. helveticus*, *L. acidophillus*, *B. lichniformis* and *B. subtilis* eliminated up to 94%, 72%, 46%, 68% and 39%, respectively, of OTA [107]; *L. plantarum*, *L. brevis* and *L. sanfrancisco* were reported to eliminate 54%, 50% and 37%, respectively, of 0.3 mg OTA/L after 24 h of incubation [108].

It is now generally accepted that OTA adsorption to the cells walls is the predominant mechanism involved in this OTA detoxification phenomenon by lactic acid bacteria (LAB). For example, adsorption effects were claimed by Turbic *et al*. [109], who found that heat and acid treated cells from two *Lactobacillusrhamnosus* strains were more effective at removing OTA from phosphate buffer solutions than viable cells. The strains removed 36% to 76% in the buffer solution (pH 7.4) after 2 h at 37 °C. Similarly, Piotrowska and Zakowska [110] verified that *L. acidophilus* and *L. rhamnosus* caused OTA reductions of 70% and 87% of 1 mg OTA/L after five days at 37 °C, and that significant levels of the OTA were present in the centrifuged bacteria cells. Other LAB (*L. brevis*, *L. plantarum* and *L. sanfranciscencis*) also produced smaller decreases on OTA (approximately 50%). Finally, Del Prete *et al.* [111] tested 15 strains of oenological LAB in order to determine the *in vitro* capacity to remove OTA, and reported *Oenococcus oeni* as the most effective, with OTA reductions of 28%. The involvement of cell-binding mechanisms was confirmed as (i) up to 57% of the OTA absorbed by the cells was recovered through methanol extraction from the bacteria pellets; (ii) crude cell-free extracts were not able to degrade OTA; and (iii) degradation products were not detected. 

Nevertheless, some authors consider that metabolism may also be involved. For example, Fuchs *et al*. confirmed that viable cells of *L. acidophilus* removed OTA more efficiently then unviable [112]. A *L. acidophilus* strain was able to decrease ≥95% the OTA in buffer solutions (pH 5.0) containing 0.5 and 1 mg OTA/L when incubated at 37 °C for 4 h. In addition, a detoxification effect was also demonstrated since pre-incubation of OTA with this strain reduced OTA toxicity to human derived liver cells (HepG2) [112]. Other *L. acidophilus* strains demonstrated only a moderate reduction in OTA contents suggesting that the effect was strain specific.

In summary, some LAB adsorb OTA by a strain specific cell-wall binding mechanism, although some undetected catabolism can also be involved. The detection of this OTA catabolism may only be possible with radiolabeled OTA. The potential of LAB as mycotoxin decontaminating agents has been reviewed [113] and which also considers *Saccharomycescerevisiae*.

#### 2.1.3. Yeasts

*S. cerevisiae* and other yeast are described erroneously in some of the literature as OTA biodegradation agents, since most of the effects detected and reported are from wall adsorption mechanisms. Several studies clearly report the adsorption effects, but others do not. *S. cerevisiae* was claimed to biodegrade 41% of 0.3 mg OTA/L after 24 h at 30 °C, but details were not provided about the mechanism involved [108]. Similarly, Böhm and co‑authors claimed that some strains degraded up to 38% of 0.05 mg OTA/L without describing any resulting degradation metabolites [107]. 

On the other hand, the adsorption of OTA by oenological *Saccharomyces* strains was demonstrated by Bejaoui and co-authors, since they verified that heat and acid treated cells could bind significantly more OTA than viable ones [114]. Viable yeast bound up to 35% and 45% of the OTA, depending on the medium and strain, while heat and acid treated cells bound a maximum of 75%. Additionally, yeast are reported to reduce OTA in alcoholic fermentation processes such as brewing or vinification. During wort fermentation, yeasts adsorbed a maximum of 21% of the added OTA [43]. Also, almost 30% of the added OTA was removed after extended contact with yeast biomass [115]. Cecchini and co-authors verified during vinification trials that up to 70% of OTA could be removed from wine and that a significant percentage of the removed OTA was found in yeast lees [116]. Adsorption assays that used several yeasts products or fractions were also carried out in order to understand and explain the mechanisms involved. Moruno and co‑authors tested the capacity of active dried yeasts and yeast lees to remove OTA from wines and reported a reduction of approximately 70% when yeast lees were used [117]. The *in vitro* biosorption of OTA by vinasse containing yeast cell walls, purified yeast *β*-glucan and dried yeast cell wall fractions was studied [118]. Dried yeast cell wall fractions were reported to be the most efficient at adsorbing OTA. Several reports explained this phenomena by relating it to yeast *β*-D-glucans [119], glucomannans [120] and mannanoligosaccharide [121]. The paper of Shetty and Jespersen reviewed the main adsorption mechanisms involved [113]. 

On the other hand, some studies emphasized the involvement of biodegradation mechanisms. For example, *Trichosporon*, *Rhodotorula* and *Cryptococcus* demonstrated an ability to biodegrade OTA through the cleavage of the amide bond and releasing OTα [122]. In this study, the most effective strain degraded up to 100% of 0.2 mg OTA/L after five hours of incubation at 35 °C. This yeast was classified subsequently as the novel species *Trichosporon mycotoxinivorans* due to its excellent ability to detoxify OTA and zearalenone [123]. The strain could counteract the OTA toxic effects to some domestic livestock. For example, when (i) introduced to the diet of broiler chickens the yeast completely blocked OTA effects to the immune system [124]; and (ii) fed to pigs with contaminated feed, higher pig body weights were observed [125]. However, a recent study recognized *T. mycotoxinivorans* as a novel human pathogen associated with cystic fibrosis and the death of a patient with histologically documented *Trichosporon* pneumonia: this obviously raises safety issues on its practical use [126]. 

A *Phaffia rhodozyma* strain was also able to degrade 90% of 7.5 mg OTA/L after 15 days at20 °C [127]. In this study, the authors were able to verify the conversion of OTA into OTα and the adsorption of OTA into viable and heat-treated cells. The involvement of a metalloproteinase similar to carboxypeptidase A in OTA biodegradation was suggested, since it was verified that the chelating agents EDTA and 1,10-phenanthroline inhibited the degradation of the mycotoxin. More recently, *Aureobasidium pullulans* was reported to degrade OTA through the hydrolysis of the amide bond since OTα was detected [128]. The use as a biocontrol agent was also assessed as a reduction of OTA in grapes and wine was reported. However, the fungus appears to be involved in human disease and this issue needs to be resolved before more general use can be recommended [129].

#### 2.1.4. Filamentous fungi

Some filamentous fungi can biodegrade OTA. Xiao and co-authors reported that *A. niger* hydrolyzed OTA and OTB [6]. *Aspergillus fumigatus*, *A. japonicus* and *A. niger* degraded 2 mg OTA/L after 10 days of incubation at 30 °C [130]. OTα was detected and further degradation into an unknown compound was observed. *Aspergillus niger* and other filamentous fungi have also shown to biodegrade OTA completely or partially, after growth in 1 mg OTA/L for six days at 25 °C [131]. OTα was detected, particularly in the assays performed with *A. niger* and other black aspergilli. An unidentified biodegradation metabolite was observed in the assays carried out with *A. ochraceus* which did not produce OTA, and some *A. wentii* strains. Additionally, *Rhizopus homothallicus*, *R. oryzae*, *R. stolonifer* and other *Rhizopus* species degraded more than 95% of 7.5 mg OTA/L after 16 days of incubation at 25 °C [132]. OTα was also detected in this study. Later, the excellent capacity of some black aspergilli to degrade OTA was confirmed: some *A. carbonarius*, *A. japonicus*, and *A. niger* strains degraded more than 80% of 2 mg OTA/L [133]. More recently and in agreement with Abrunhosa *et al*. [131], the capacity of *Botrytis cinerea* to degrade OTA was confirmed with reductions of 24.2% to 26.7% [134]. This provided an explanation for the low OTA contamination of noble rot and late-harvest wines. The white rot fungus *Pleurotus ostreatus* could degrade OTA (77%) and OTB (97%) when growth on contaminated barley by solid state fermentation, with OTα being detected from OTA biodegradation [135]. *Rhizopus japonicus* and *Phanerochaete chrysosporium* were also shown to biodegrade OTA to the lesser extents of 38% and 36%, respectively.

#### 2.1.5. Plant cell cultures

Finally, it was shown that cell cultures of wheat, maize, tomato, soybean, sweet potato and other plants completely transform OTA [136].

### 2.2. Enzymes which degrade ochratoxin A

**Figure 3:**
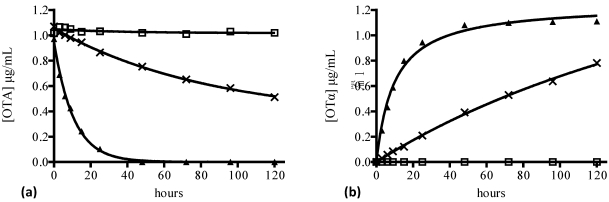
Ochratoxin A biodegradation by carboxypeptidase Y (CPY) from *S. cerevisiae*. (**a**) OTA detected in assays *versus* time. (**b**) OTα detected in the same assays *versus* time.-×- assay with 0.1 mg/mL of CPY at pH 5.6, 37 °C; -▲- assay with 0.5 mg/mL of CPA (control) at pH 7.5, 37 °C, -□- assay with no enzyme at pH 5.6, 37 °C (blank). Material, methods were as reported in [140].

Several enzymes may be involved in the microbiological degradation of OTA. However, little information is available and very few have been purified and characterized. The first reported protease able to hydrolyze OTA was carboxypeptidase A (CPA) (EC 3.4.17.1) from bovine pancreas [137]. Subsequently, a screening study which included several commercial hydrolases, verified that a crude lipase product from *A. niger* was able to hydrolyze OTA via the amide bond [138]. The enzyme was purified by anion exchange chromatography and was demonstrated to cleave OTA and p‑nitrophenyl palmitate, a specific lipase substrate. Several proteolytic preparations were also studied, which were involved in the hydrolysis of OTA to OTα. These included protease A from *A. niger*, pancreatin from porcine pancreas and to a lesser extent, Prolyve PAC from *A. niger *[139]. Additionally, the production and purification of an *A. niger* cell-free crude enzyme preparation that demonstrated a significant capacity to cleave the amide bond of OTA was reported. The OTA-degrading enzyme involved was purified by anion exchange chromatography and characterized [140]. This enzyme showed higher OTA-degrading activity then CPA at pH 7.5 and 37 °C, and was inhibited by EDTA, which is a specific inhibitor of metalloproteases. In subsequent studies [141], it was found that carboxypeptidase Y (CPY) (EC 3.4.16.1) from *S. cerevisiae* is also able to hydrolyze OTA with optimal activity at pH 5.6 and 37 °C (Figure 3). However, the specific activity of CPY is very low as indicated by the OTA hydrolyzation reaction being very slow. Nevertheless, after five days of incubation, CPY converted 52% of the OTA present in the reaction assay into OTα. This activity is sufficient to reduce significantly levels of OTA during wine or beer fermentation, since these processes take several days to complete. Hence, a biodegradation pathway is possible for *S. cerevisiae *in addition to the OTA adsorption phenomenon. It is necessary to consider that CPY is a vacuolar exopeptidase where OTA enters the yeast cells before it is catabolized. However, the *S. cerevisiae* wall-binding properties can make difficult OTA uptake. Further information concerning the enzymes which are able to hydrolyze OTA is presented in Table 4.

**Table 4:** Reported pure enzymes and enzyme formulations that hydrolyze OTA.

Commercial name	Origin	Main activity	Supplier	Reference
Carboxypeptidase A	*Bovinus bovis*	exopeptidase	Boehringer	[142]
Carboxypeptidase Y	*Saccharomyces cerevisiae*	exopeptidase	Sigma	not reported
Lipase	*Aspergillus niger*	Lipase	Amano Inc.	[138]
Enzyme preparations	*-*	Proteolysis	-	[143]
Protease A	*Aspergillus niger*	Acid protease	Amano Inc.	[139]
Prolyve PAC	*Aspergillus niger*	Acid protease	Lyven	[139]
Pancreatin 4XNF-P211P	Porcine pancreas	Amylase, lipase and protease	Biocatalysts	[139]
Crude extract	*Aspergillus niger*	OTA-hydrolase	-	[140]

- data not available.

## 3. Conclusions

A significant proportion of the world food crops is contaminated with mycotoxins, and safer methods for decontamination and prevention are required. Microorganisms and enzymes could be a practical way to reduce the concentrations and to avoid the toxic effects via bioremediation. OTA biodegradation occurs with various microorganisms. However, in some cases it is not clear if biodegradation occurred or if adsorption mechanisms were involved. OTA biodegradation is difficult to demonstrate, especially if an extensive catabolization occurs during the assays, *i.e.*, if OTA is broken down into such small units that they are metabolized by the microbiological agent. In these situations, only the use of radiolabeled OTA will clarify the situation, although this approach has not been undertaken. 

In addition, the identification of the enzymes involved is complex since it is necessary to employ the appropriate pH, temperature, ionic-strength and time in order to allow the catalytic reaction to occur, and to measure the reaction. Amide bond hydrolysis releases OTα that is detectable by the methods used to detect OTA and so this is an advantage. However, alternative biodegradation pathways can lead to other compounds that are more difficult to detect. Finally, it is important that more OTα and OP‑OTA toxicology studies are undertaken to ensure the safety of the degradation products.

The use of microorganisms such as LAB and* S. cerevisiae* present great advantages since they have an historical and extensive use in the food industry. In addition, they have well-known probiotic properties. On the other hand, the high purification costs of enzymes can render their practical application inviable and crude enzyme extracts may be a viable alternative. In conclusion, the bioremediation of OTA contaminated foods with microorganisms and enzymes will lead to safer food by using these environmentally-sound processes.
